# Primary Headache Is Related to Reduced Health-Related Quality of Life in Children with Epilepsy

**DOI:** 10.3390/healthcare12040426

**Published:** 2024-02-07

**Authors:** Katharina Schiller, Veronika Schiller, Aline Kortas, Gabriele Unterholzner, Sabine Raffler, Mareike Schimmel, Markus Rauchenzauner

**Affiliations:** 1 Department of Pediatrics, Hospital Group Kaufbeuren-Ostallgäu, 87600 Kaufbeuren, Germany; 2 Department of Neurology and Neurosurgery, Montreal Neurological Hospital and Institute, Montreal, QC H3A2B4, Canada; 3 Department of Pediatrics, Medical University Innsbruck, 6020 Innsbruck, Austria; 4 Faculty of Medicine, Paracelsus Medical University, 5020 Salzburg, Austria; 5 Pediatrics and Adolescent Medicine, Faculty of Medicine, University of Augsburg, 86156 Augsburg, Germany

**Keywords:** children, headache, epilepsy, quality of life, migraine, tension-type headache

## Abstract

Headache is a frequent comorbidity in patients with epilepsy. Data are sparse regarding the distribution of headache types in children with epilepsy (CWE). We aimed to assess the prevalence of primary headache types and their influence on health-related quality of life (QoL) in CWE. CWE filled out a validated headache questionnaire to assess migraine (MIG), tension-type headache (TTH), trigeminal–autonomic cephalalgia (TAC), or, if the criteria were not fulfilled, non-classifiable headache (NCH). QoL was measured using both patient and parent versions of a validated questionnaire. Of 119 CWE (59 female; 11.5 ± 3.1 y), headache was found in 46 (38.7%). Sixteen (34.8%) patients showed MIG, 9 (19.6%) patients TTH, and 21 (45.7%) patients described NCH. More girls reported headache (*χ*^2^ = 5.4, *p* = 0.02) when compared to boys. Overall, QoL was reduced in patients with headache from both the patients’ and parents’ points of view (70.8% [39.6; 87.5] vs. 77.0% [46.9; 95.8], *p* = 0.002; 71,9% [33.3; 87.5] vs. 78,1% [54.2; 95.8], *p* = 0.003). Headache is common among CWE with MIG as the most prevalent primary headache type and higher rates in female patients. Importantly, patients and their parents perceive a reduced overall QoL when suffering from headache.

## 1. Introduction

Among the most common comorbidities of patients with epilepsy is headache [1]. Besides a shared common pathophysiology between epilepsy and headache, in particular migraine [2], genetic disposition also seems to play an important role [2,3]. Primary headache types are described as headaches without an apparent underlying cause [4] and the main categories are, according to the International Classification of Headache Disorders (ICHD-3), migraine (MIG), tension-type headache (TTH), trigeminal–autonomic cephalgia (TAC), and neuralgia [5].

MIG is described in adults as unilateral, pulsating headaches of moderate or severe intensity, which can be intensified by physical activity and can occur with or without an aura. For children, these criteria were modified and include bilateral headache, lasting 2 to 72 h, along with nausea and/or vomiting and an additional two of five other associated symptoms (phototopia, phonophobia, difficulty thinking, lightheadedness, fatigue) [6]. The prevalence of MIG in the general pediatric population varies between 5–30% in preschool children and adolescents, and symptoms can vary with the age of the child [7,8,9,10]. Various studies have shown an association between epilepsy and MIG, which share common clinical features [11]. In a pediatric headache center, the risk of epilepsy in children with MIG was 3.2 times higher than in children with TTH [12]. Two antiseizure medications, topiramate and valproic acid, are also approved for MIG prophylaxis [13,14,15,16]. TTH, as the second category of primary headache types, is typically characterized by a bilateral headache which is non-pulsating and a pressing pain with mild–moderate intensity [5]. TTH is frequently found in the general population and a systematic review in children showed a prevalence of 17% [10]. The third category of primary headache types is TAC, which appears unilaterally in the orbital, supraorbital, or temporal region with intense to very intense pain [5]. The prevalence of TAC is very low, affecting roughly 0.1% of people, especially men [17].

Children with epilepsy (CWE) are at risk of showing a reduced overall health-related quality of life (QoL) and reduced family activities or impacted mental health [18,19]. They are at risk of many comorbidities and must also cope with the stress of managing seizures [20]. This burden may also impact social aspects of their lives [21]. There is some evidence that children with episodic MIG and also adults suffering from MIG experience lower QoL than healthy controls [22,23]. Currently, it is unknown whether comorbid headache might lead to a reduction of QoL in children with epilepsy.

Data are sparse regarding the distribution of primary headache types in CWE. Therefore, the goal of the present study was to assess the prevalence of primary headache types (MIG, TTH, and TAC) in CWE, as well as gender differences. Additionally, we aimed to investigate their influence on health-related QoL from both the child’s and parent’s perspectives.

## 2. Materials and Methods

In this prospective bi-center study, patients with diagnosed epilepsy visiting the seizure outpatient clinic of the Children’s Hospital Kaufbeuren (March 2020–December 2021) and the University Children’s Hospital Augsburg (March 2019–December 2019) were included. Written informed consent was obtained from all patients and parents. This study was performed in accordance with the Declaration of Helsinki and approved by the Research Ethics Board of the Ludwig-Maximilians-University Munich (18–515, 27 November 2018).

The study population consisted of *n* = 119 children (59 female, 60 male) diagnosed with epilepsy by a pediatric neurologist [24]. Seventy-six patients were included at the Children’s Hospital Kaufbeuren and 43 patients at the University Children’s Hospital Augsburg. Study participants fulfilled the criteria of (i) a documented diagnosis of epilepsy and (ii) age between 7 and 17 years to enable completion of the validated questionnaires. Children had to be able to fill out the questionnaires. Therefore, patients with severe cognitive impairment were not included in this study. Data were obtained during outpatient visits. First, patients were asked if they experienced headaches. If they reported having headaches, they were given the headache questionnaire, the self-developed questionnaire with epilepsy-related and sociological items, and the QoL questionnaire. If patients denied having headaches, they skipped the headache questionnaire and filled out the self-developed questionnaire with epilepsy-related and sociological items as well as the patient version of the QoL questionnaire. For all patients, one or both parents filled out the parent version of the QoL questionnaire.

### 2.1. Headache Questionnaire

To assess the different headache types, we used a validated questionnaire designed to differentiate between MIG, TTH, and TAC [25] based on the classification criteria of the International Headache Society (ICHD-2) [26]. A fourth category described non-classifiable headache (NCH) if not all criteria for a main headache type were fulfilled. There were three blocks for MIG, TTH, and TAC; the first two blocks for MIG and TTH consisted of seven items respectively, with two additional items to differentiate between MIG with or without aura. TAC was assessed by six items. Each item (e.g., Do you feel a pulsating headache?) had to be answered with ‘yes’ or ‘no’. Following each block, the total days per month with this kind of headache were reported. This allowed for differentiation between chronic headache (15 days or more) and episodic headache (14 days or less). At the end, the following headache types were determined: MIG with aura, MIG without aura, TTH, TAC, and the combinations of MIG + TTH, MIG + TAC, and TTH + TAC. If the criteria for any of these specific headache types were not fulfilled, the headache was described as non-classifiable (NCH).

### 2.2. Epilepsy-Related and Sociological Data

In a self-developed questionnaire, age, sex, and data regarding epilepsy such as duration of epilepsy and seizure freedom in the last 12 months (yes/no) were assessed. If patients reported having headaches, they were further asked whether the headache occurred together with a seizure (before = preictal/during = ictal/after = postictal). We added items regarding media consumption (television, computer, gaming console: h/week) and whether the child is performing physical activity (sports: yes/no; if yes: h/week).

### 2.3. Quality of Life Questionnaire

To assess health-related QoL, we administered the questionnaire ‘Kinder Lebensqualität Fragebogen’ (KINDL-R) measuring QoL in children and adolescents between 7–17 years [27,28]. Additionally, the parent’s version was filled out by the accompanying mother, father, or both parents. This questionnaire consisted of 24 items measuring the average feelings and experiences during the previous week using a five-point Likert scale (e.g., during the past week, I felt strong and full of energy; 1 = never to 5 = always). A total score and the following six subscales could be calculated: physical and emotional wellbeing, self-esteem, family, friends, and school. Standardized values are reported on a scale from 0 to 100 with 100 representing the maximum QoL.

### 2.4. Statistics

Data were tested for normality using the Kolmogorov–Smirnov test and are reported as absolute numbers and percentages in parentheses. Due to non-normally distributed data, groups were compared using the Mann–Whitney U-test. Contingency tables and Pearson’s Chi-squared test (*χ*^2^-test) were computed where applicable. A *p*-value ≤ 0.05 was considered statistically significant. Statistical analyses were performed with the Statistical Package for Social Sciences for Mac (SPSS Inc., Chicago, IL, USA, version 28.0).

## 3. Results

In total, 119 children with diagnosed epilepsy (59 female; 11.5 ± 3.1 years old) were included in this study. Duration of epilepsy was, at the time of study inclusion, 3.9 ± 3.5 years. Overall, 39 patients (32.8%) had focal, 56 patients (47.0%) generalized, 11 combined focal and generalized (9.3%), and 13 patients (10.9%) were classified with unknown epilepsy. Ninety-five patients (79.8%) had genetic, twelve patients (10.1%) structural, and twelve patients (10.1%) unknown etiology. Within the whole study population, 63 patients (52.9%) were under antiseizure medication monotherapy, 29 (24.4%) were undergoing polytherapy, and 27 (22.7%) had been given no medication at the time of study inclusion.

### 3.1. Distribution of Headache

**Figure 1:**
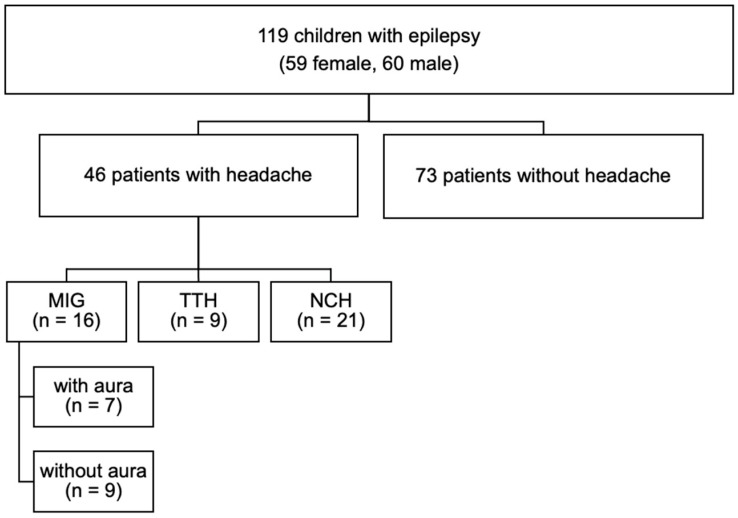
Flowchart presenting headache distribution (MIG = migraine, TTH = tension-type headache, NCH = non-classifiable headache).

Out of 119 epilepsy patients, 46 patients (38.7%) reported suffering from headache. Baseline characteristics are presented in Table 1. Among the 46 patients with headache, 16 (34.8%) fulfilled the criteria for MIG, of which 9 had MIG without aura and 7 MIG with aura. A total of 9 (19.6%) patients reported TTH and the headache of 21 (45.7%) patients was categorized as non-classifiable (NCH) (Figure 1). No patients reported TAC or any combination of primary headache types. Altogether, headache was classified as chronic (≥15 days per month) in two (4.3%) patients, with one reporting chronic MIG without aura and one reporting chronic MIG with aura.

### 3.2. Headache in Relation to Epilepsy Type

**Figure 2:**
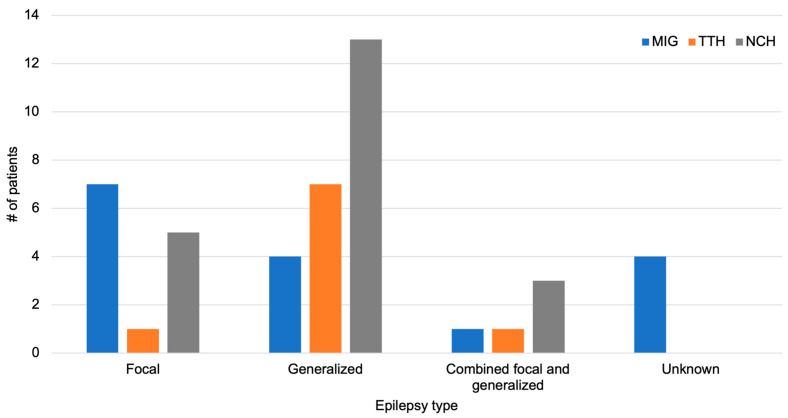
Primary headache type in relation to different epilepsy types.

Of the 46 patients reporting headache, 13 patients (28.2%) had focal epilepsy, 24 (52.1%) had generalized epilepsy, 5 (10.9%) had combined focal and generalized epilepsy, and 4 (8.7%) had unknown epilepsy. The distribution of the primary headache types in relation to the type of epilepsy is depicted in Figure 2. The association between primary headache type and type of epilepsy was significant (*χ*^2^ = 14.3, *p* = 0.027). Patients with focal epilepsy mostly reported MIG (n = 7 out of 13) whereas patients with generalized epilepsy most often reported NCH (n = 13 out of 24).

### 3.3. Headache within Female and Male Patients

**Figure 3:**
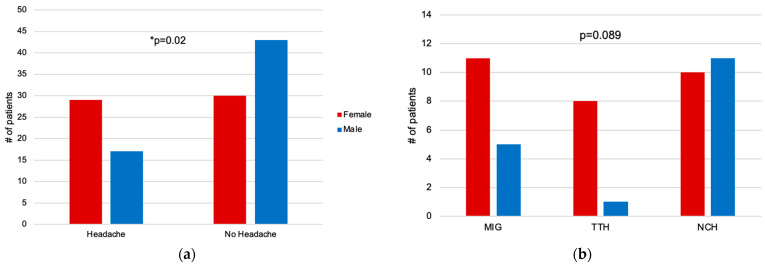
(**a**) Number of female and male patients with and without headache; (**b**) Distribution of primary headache types among female and male patients. Significant results (*p* < 0.05) are indicated by a star.

Female patients had a higher proportion of headache than male patients. A total of 29 of 59 (49.2%) females and 17 of 60 (28.3%) males experienced headache (Figure 3a). This difference was statistically significant (*χ*^2^ = 5.4, *p* = 0.02).

Of the 29 female patients reporting headaches, 11 (37.4%) showed MIG, 7 without aura and 4 with aura. A total of 8 (27.6%) patients reported TTH and 10 (34.5%) NCH. Of 17 male patients with headache, 5 (29.4%) had MIG, 2 without aura and 3 with aura. Only 1 (5.9%) patient reported TTH whereas 11 (64.7%) described NCH. There was no significant difference between female and male patients regarding the distribution of different headache types (*χ*^2^ = 4.9; *p* = 0.089) (Figure 3b). The two patients reporting chronic MIG were male patients whereas no female patients reported chronic headache (≥15 days per month or more).

### 3.4. Seizure-Associated Headache

Out of 46 children with headache, 22 (47.8%) described experiencing seizure-associated headaches. Isolated postictal headache was the most frequently reported seizure-associated headache; this was reported by 12 patients and was followed by isolated preictal headache, which was described by 4 patients. No patients reported isolated ictal headache. Five patients had preictal and postictal headache, and one patient reported preictal and ictal headache.

### 3.5. Headache and Seizure Freedom

In the total study population of 119 CWE, 88 (73.9%) were seizure-free for the last 12 months and 31 (26.1%) were not. The association between headache and seizure freedom was statistically significant (*χ*^2^ = 6.6, *p* = 0.01). There was a higher proportion of children with headache when they were not seizure-free.

### 3.6. Headache and Sociological Variables

Regarding media consumption, there was no significant difference in patients with headache and without headache (2 h [0; 15] vs. 2 h [0; 10] *p* = 0.473). Further, there was no significant association between headache and physical activity (yes/no) and no significant difference between patients with and without headache with regards to the duration of physical activity per week (4 h [1; 10] vs. 5 h [1; 20], *p* = 0.226).

### 3.7. Quality of Life

**Figure 4:**
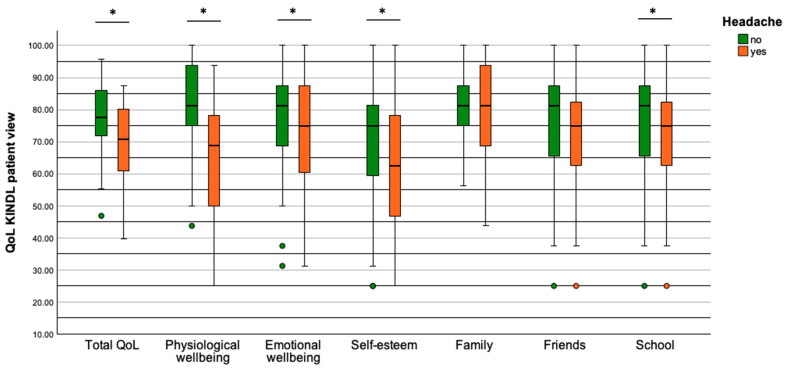
QoL from the patient’s view in terms of overall QoL and six subscales. Significant reductions were seen in QoL in patients with headache in terms of overall QoL, physiological wellbeing, emotional wellbeing, self-esteem, and school. Significant results (*p* < 0.05) are indicated by a star.

Patients with headache reported a reduced total QoL compared to patients without headache (70.8% [39.6; 87.5] vs. 77.6% [46.9; 95.8], *p* = 0.002, Figure 4). Furthermore, they described a reduced QoL in the subscales physical wellbeing (68.8% [25.0; 93.8] vs. 81.3% [43.8; 100.0], *p* ≤ 0.001), emotional wellbeing (75.0% [31.3; 100.0] vs. 81.3% [31.3; 100.0], *p* = 0.038), self-esteem (62.5% [25.0; 100.0] vs. 75.0% [25.0; 100.0], *p* = 0.043), and school (68.8% [25.0; 93.8] vs. 75.0% [12.5; 100.0], *p* = 0.004). The subscales family and friend were not significantly different between patients with and without headache (family: *p* = 0.50; friends: *p* = 0.36).

**Figure 5:**
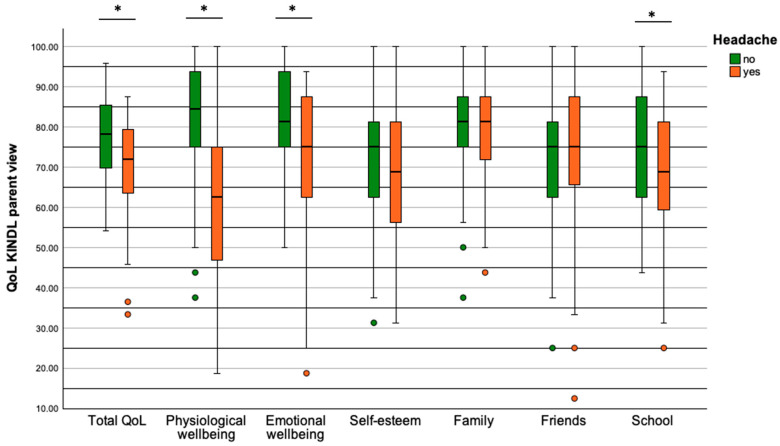
QoL from the parent’s view in terms of overall QoL and six subscales. Significant reductions were seen in QoL in patients with headache in terms of overall QoL, physiological wellbeing, emotional wellbeing, and school. Significant results (*p* < 0.05) are indicated by a star.

Parents rated their children equally in the parent version of the QoL questionnaire and report a reduced overall QoL for patients with headache compared to patients without headache (71.9% [33.3; 87.5] vs. 78.1% [54.2; 95.8], *p* = 0.003; Figure 5). Similarly, they saw the QoL of their children decrease in the subscales physical wellbeing (62.5% [18.8; 93.8] vs. 84.4% [37.5; 100.0], *p* = 0.002), emotional wellbeing (75.0% [18.8; 93.8] vs. 81.3% [50.0; 100.0], *p* = 0.002), and school (68.8% [25.0; 93.8] vs. 75.0% [43.75; 100.0], *p* = 0.002). The parent-rated subscales self-esteem (*p* = 0.20), family (*p* = 0.36), and friends (*p* = 0.87) were not significantly different between patients with and without headache.

### 3.8. Quality of Life and Sociological Variables

Media consumption was negatively correlated with overall QoL (r = −0.247, *p* = 0.012), emotional wellbeing (r = −0.215, *p* = 0.029), and friends (r = −0.211, *p* = 0.033) from the patients’ views whereas there was no correlation regarding physical wellbeing (*p* = 0.084), self-esteem (*p* = 0.302), family (*p* = 0.379), and school (*p* = 0.087). Additionally, the correlation between QoL and media consumption from the parents’ views was not significant (total QoL *p* = 0.787, physical wellbeing *p* = 0.442, emotional wellbeing *p* = 0.719, self-esteem *p* = 0.371, family *p* = 0.318, friends *p* = 0.579, school *p* = 0.129). From patients’ views, there was a borderline significant enhancement in QoL when they engaged in physical activities, with higher overall QoL (physical activity: 79.2% [39.6; 95.8] vs. no physical activity: 2.9% [39.6; 89.6], *p* = 0.066) as well as in the subscale friends (75.0% [25.0; 100.0] vs. 71.9% [25.0; 100.0] *p* = 0.057). From parents’ views, QoL did not differ between both groups (total QoL *p* = 0.132, physical wellbeing *p* = 0.131, emotional wellbeing *p* = 0.337, self-esteem *p* = 0.819, family *p* = 0.657, friends *p* = 0.286, school *p* = 0.211). The amount of physical activity from the patients’ and parents’ views was not correlated with QoL and the subscales (total QoL *p* = 0.436, physical wellbeing *p* = 0.569, emotional wellbeing *p* = 0.289, self-esteem *p* = 0.921, family *p* = 0.786, friends *p* = 0.568, school *p* = 0.324).

## 4. Discussion

In this study, primary headache and QoL were prospectively assessed in a study population of children with diagnosed epilepsy. The main findings indicate that headache is a frequent comorbidity in pediatric epileptic patients with MIG being the most common primary headache type and female patients being more often affected. Further, headache not only reduces the health-related QoL of CWE but also impacts several subscales such as physical wellbeing, emotional wellbeing, self-esteem, and school.

In our study population, headache was a common comorbidity among epilepsy patients with more than one-third of children indicating headaches, which is in line with previous studies [29]. Approximately one-third of children with headaches reported having MIG, which is slightly higher than the one-fourth reported by Kelley et al. [30] and lower than the 43.5% found in Yamane et al. [31]. With regards to the whole study population, 13% of CWE showed MIG which is greater than what was described in a meta-analysis involving the general pediatric population [10]. The higher frequency of MIG in epileptic patients may be explained by shared pathophysiology and genetic disposition [11]. Studies have suggested that the underlying pathophysiology of MIG and epilepsy might be explained by cortical hyperexcitability [32]. There is further the possibility of a genetic link between epilepsy and MIG which could explain the shared pathophysiology [33]. Due to shared clinical symptoms, MIG attacks and epileptic seizures might be confounded, especially in children [11]. According to ICHD-3 [5], an epileptic seizure preceded by a MIG attack is classified as ‘migralepsy’. This is a rare event seen in patients with MIG with aura, which is not included in the criteria for epileptic seizures by the International League Against Epilepsy [34]. Only a very small subset of the patients in this study indicated chronic MIG. The prevalence of MIG is higher during adolescence than during childhood and was also reported to have increased during the coronavirus disease (COVID-19) pandemic [35]. This may also contribute to the high prevalence of MIG in our study cohort.

TTH was less frequent than MIG, with every fifth child experiencing headaches indicating TTH. Findings in adult patients with epilepsy have showed that MIG and TTH might be the most frequent primary headache types with a similar prevalence of around 33% [29,36]. The lower incidence of TTH in children and a high prevalence of NCH in our study population might suggest that part of the NCH in younger age groups may develop into a more defined primary headache type in adulthood. In the whole study population, no child reported the primary headache type TAC or any combination of primary headache types which we previously found in a small subset of our epileptic adult population [36]. Epidemiological data on less common headache types such as TAC are also lacking in the general pediatric population [10]. The type of epilepsy was further associated with the primary headache type. While focal epilepsy patients reported MIG more frequently, patients with generalized epilepsy reported mostly NCH followed by TTH.

The prevalence of headache was higher in female than male children, with female patients more often reporting MIG and TTH-type headaches. A higher prevalence of girls reporting headaches was also found in the general pediatric population [10]. However, in the context of epilepsy, the results are inconsistent. Some studies have found gender differences, reporting higher numbers in female patients [12,36], while others have found no difference [31]. Chronic headache was rare in our population, reported only by two male patients with chronic MIG. This is comparable to results found in children with chronic MIG who often show previous episodic MIG [7]. While it was previously shown that there is a tendency for girls to develop chronic MIG [7], our findings are in contrast to this pattern. However, this might be due to our smaller sample size.

Seizure-associated headache was frequent in CWE and most children described postictal headache followed by preictal headache. This is in line with previous findings in children as well as adults with epilepsy [37,38,39]. In children with drug-resistant epilepsy, MIG was especially found as postictal headache whereas TTH was more common among children with non-drug-resistant epilepsy [40]. Furthermore, there was an association between seizure freedom and headache with a higher proportion of seizure freedom in the subgroup of children without headache.

Patients with headaches were found to have a reduced QoL in comparison to patients without headaches. This was not only evident from the patients’ perspectives but also from the parents’ views on overall QoL as well as on several other domains such as physical wellbeing, emotional wellbeing, self-esteem, and school. Epilepsy treatment and disease management can be very challenging for patients and their families, and the physiological and psychological burden is high [41,42]. Headache seems to have an additional negative impact on CWE, not only physically and emotionally but also academically. Therefore, it is of importance in everyday practice to recognize headaches as a significant influencing factor in disease management and in the patient’s QoL, and to offer tailored support for children and their families.

Finally, we examined the association between sociological factors, such as media consumption and physical activity of patients and the incidence of headache, as well as QoL. While both factors were not related to the existence of headache, media consumption was negatively correlated with QoL from the patients’ views. Contrarily, physical activity tended to increase QoL. The benefit of physical activity on QoL is also known in the general pediatric population [43]. Reducing media consumption and increasing physical activity could therefore counterbalance the detrimental effect of headache on QoL in CWE.

There are some limitations to our study. Headaches were only assessed once, and the sample size limits the generalizability of the results. However, the data were collected prospectively in two different pediatric neurology clinics. Future studies should take family history of headache into consideration. Additionally, the use of a headache diary over a longer time period may allow for more distinct analysis. This would also allow the more detailed study of headache onset, intensity, and frequency. Further, the patients were followed up in the epilepsy clinic and other diseases might be more easily detected than in a child who is not regularly seen in the hospital [44]. However, the known link between epilepsy and MIG suggests that children with epilepsy experience MIG more often than their peers without epilepsy. Finally, psychiatric disorders such as anxiety or depression, which are also present in children with epilepsy, can have a negative impact on QoL [45]. These factors were not considered in our study so the results should be interpreted with caution.

## 5. Conclusions

In CWE, headache is an important comorbidity and MIG appears to be the most common primary headache type. Female patients reported comorbid headache more often than male patients. From the patients’ and parents’ views, a reduced QoL was indicated when the child was affected by headaches. This should be taken into consideration when treating CWE as headache seems to impact patients in addition to the burden of epilepsy itself.

## Figures and Tables

**Table 1:** Baseline characteristics of the whole study cohort and patients with primary headache.

	All Patients with Epilepsy	Patients with Epilepsy and Primary Headache
N=	119	46
Age, y (mean ± SD)	11.5 ± 3.1	11.3 ± 3.2
Sex (female/male)	59/60	29/17
Epilepsy duration, y (mean ± SD)	3.9 ± 3.5	4.6 ± 4.3
Types of epilepsy, n (%)		
Focal	39 (32.8%)	13 (28.3%)
Generalized	56 (47.0%)	24 (52.1%)
Combined focal and generalized	11 (9.3%)	5 (10.9%)
Unknown	13 (10.9%)	4 (8.7%)
Etiology		
Genetic	95 (79.8%)	35 (76.1%)
Structural	12 (10.1%)	6 (13.0%)
Unknown	12 (10.1%)	5 (10.9%)
Antiseizure medication, n (%)		
Monotherapy	63 (52.9%)	24 (52.2%)
Polytherapy	29 (24.4%)	14 (30.4%)
No medication	27 (22.7%)	8 (17.4%)

## Data Availability

The data presented in this study are available on request from the corresponding author.
